# Serotonin Receptor Gene Polymorphisms Are Associated with Cerebrospinal Fluid, Genetic, and Neuropsychological Biomarkers of Alzheimer’s Disease

**DOI:** 10.3390/biomedicines10123118

**Published:** 2022-12-02

**Authors:** Mirjana Babić Leko, Matea Nikolac Perković, Ena Španić, Dubravka Švob Štrac, Nikolina Pleić, Željka Vogrinc, Ivana Gunjača, Dora Bežovan, Gordana Nedić Erjavec, Nataša Klepac, Fran Borovečki, Tatijana Zemunik, Nela Pivac, Patrick R. Hof, Goran Šimić

**Affiliations:** 1 Department of Neuroscience, Croatian Institute for Brain Research, University of Zagreb Medical School, 10000 Zagreb, Croatia; 2 Department of Medical Biology, School of Medicine, University of Split, 21000 Split, Croatia; 3 Department of Molecular Medicine, Institute Ruđer Bošković, 10000 Zagreb, Croatia; 4 Laboratory for Neurobiochemistry, Department of Laboratory Diagnostics, University Hospital Centre Zagreb, 10000 Zagreb, Croatia; 5 General Hospital Zabok, 49210 Zabok, Croatia; 6 Department of Neurology, University Hospital Centre Zagreb, 10000 Zagreb, Croatia; 7 Nash Family Department of Neuroscience, Friedman Brain Institute, Ronald M. Loeb Center for Alzheimer’s Disease, Icahn School of Medicine at Mount Sinai, New York, NY 10029, USA

**Keywords:** Alzheimer’s disease, 5-hydroxytryptamine (serotonin), 5HT receptors, biomarkers, cerebrospinal fluid, Mini-Mental State Examination, apolipoprotein E

## Abstract

A decrease in serotonergic transmission throughout the brain is among the earliest pathological changes in Alzheimer’s disease (AD). Serotonergic receptors are also affected in AD. Polymorphisms in genes of serotonin (5HT) receptors have been mostly associated with behavioral and psychological symptoms of dementia (BPSD). In this study, we examined if AD patients carrying different genotypes in *5HTR1B* rs13212041, *5HTR2A* rs6313 (T102C), *5HTR2C* rs3813929 (−759C/T), and *5HTR6* rs1805054 (C267T) polymorphisms have a higher risk of faster disease progression (assessed by neuropsychological testing), are more prone to develop AD-related pathology (reflected by levels of cerebrospinal fluid [CSF] AD biomarkers), or have an association with an apolipoprotein E (*APOE*) haplotype. This study included 115 patients with AD, 53 patients with mild cognitive impairment (MCI), and 2701 healthy controls. AD biomarkers were determined in the CSF of AD and MCI patients using enzyme-linked immunosorbent assays (ELISA), while polymorphisms were determined using either TaqMan SNP Genotyping Assays or Illumina genotyping platforms. We detected a significant decrease in the CSF amyloid β_1–42_ (Aβ_1–42_) and an increase in p-tau_181_/Aβ_1–42_ ratio in carriers of the T allele in the *5HTR2C* rs3813929 (−759C/T) polymorphism. A significantly higher number of *APOE* ε4 allele carriers was observed among individuals carrying a TT genotype within the *5HTR2A* T102C polymorphism, a C allele within the *5HTR1B* rs13212041 polymorphism, and a T allele within the *5HTR6* rs1805054 (C267T) polymorphism. Additionally, individuals carrying the C allele within the *5HTR1B* rs13212041 polymorphism were significantly more represented among AD patients and had poorer performances on the Rey–Osterrieth test. Carriers of the T allele within the *5HTR6* rs1805054 had poorer performances on the MMSE and ADAS–Cog. As all four analyzed polymorphisms of serotonin receptor genes showed an association with either genetic, CSF, or neuropsychological biomarkers of AD, they deserve further investigation as potential early genetic biomarkers of AD.

## 1. Introduction

The serotonergic system is severely affected in Alzheimer’s disease (AD) [1,2,3,4]. Indeed, serotonin (5-hydroxytryptamine, 5HT) is an indoleamine released by serotonergic neurons located in the brainstem raphe nuclei. These nuclei are divided into a rostral (B5–B9) and a caudal (B1–B3) raphe group [5,6,7,8]. The main serotonergic nucleus, the dorsal raphe nucleus (DRN, B7–B9), projects throughout the cerebral cortex (reviewed in [9]). Moreover, 5HT binds to serotonergic receptors. There are seven types of serotonergic receptors, with several subtypes (5HTR_1A-F_, 5HTR_2A-C_, 5HTR_3A-E_, 5HTR_4_, 5HTR_5A-B_, 5HTR_6_, 5HTR_7_). All 5HT receptors, except for 5HTR_3_, a ligand-gated ion channel, are G-protein-coupled receptors [10,11].

Loss of serotonergic innervation of the hippocampus and neocortex [2,11,12,13], decrease in the levels of 5HT and 5HT metabolites [14,15], and accumulation of AD pathological changes in serotonergic nuclei [16] have all been reported in AD. In addition, the loss of 5HT receptors and 5HT receptor binding was observed in AD [17,18,19]. Polymorphisms in genes for 5HT receptors have been associated with behavioral and psychological symptoms of dementia (BPSD) [20,21,22,23,24,25,26]. The *5HTR2A* rs6313 (T102C) and *5HTR6* rs1805054 (C267T) polymorphisms were previously associated with AD, while the association of the *5HTR1B* rs13212041 and *5HTR2C* rs3813929 (−759C/T) polymorphisms with AD was not previously noticed. This study assessed whether the levels of cerebrospinal fluid (CSF) AD biomarkers, scores on neuropsychological tests, and genetic biomarkers of AD (apolipoprotein E (*APOE*) haplotype) differ between AD patients with various *5HTR1B* rs13212041, *5HTR2A* rs6313 (T102C), *5HTR2C* rs3813929 (−759C/T), and *5HTR6* rs1805054 (C267T) polymorphisms. CSF AD biomarkers serve as endophenotypes of AD as they reflect AD pathological changes [27], while neuropsychological tests show potential in monitoring disease progression [28]. CSF amyloid β_1–42_ (Aβ_1–42_) is an index of amyloid plaque deposition [29], phosphorylated tau proteins reflect neurofibrillary tangles [30], and total tau (t-tau) and visinin-like protein 1 (VILIP-1) are markers of neurodegeneration [31,32]. We tested the potential of such polymorphisms as genetic biomarkers of AD and certain genotypes as representing a genetic predisposition to develop AD-related pathologies and faster disease progression.

## 2. Materials and Methods

### 2.1. Subjects

This study included 168 patients recruited at the University Hospital Center Zagreb and 2701 healthy controls (HC) from the “10,001 Dalmatians project” (part of the Croatian Biobank program [33]). AD was diagnosed using the criteria of the National Institutes on Aging–Alzheimer’s Association (NIA–AA) [34], while mild cognitive impairment (MCI) was diagnosed using the criteria of Petersen et al. [35] and Albert et al. [36]. Participants gave informed consent for participation in the study, and the Central Ethical Committee of the University of Zagreb Medical School (case no. 380-59-10106-18-111/126, class 641-01/18-02/01 from 20 June 2018), Ethical Committee of the Clinical Hospital Center Zagreb (case no. 02/21 AG, class 8.1-18/82-2 from 24 April 2018), and Ethical board of the University of Split, School of Medicine (case no. 2181-198-03-04-14-0031 and 2181-198-03-04-19-0022) approved all procedures. Additionally, all procedures performed within this study were in accord with the Helsinki Declaration [37]. Patients underwent neurological examination, examination of thyroid function, and serology for syphilis and Lyme disease. The levels of vitamin B12 and B9 (folic acid) were also determined in each patient. Table 1 summarizes information on biomarkers and demographic data, while Table 2 summarizes information on determined *5HTR* and *APOE* genotypes.

### 2.2. Neuropsychological Testing

Patients were neuropsychologically tested using the Mini-Mental State Examination (MMSE), the Alzheimer’s Disease Assessment Scale–cognitive subscale (ADAS–Cog), the Clock Drawing Test (CDT), the Rey–Osterrieth complex figure test (ROCFT), and the Visual Association Test (VAT).

### 2.3. Analysis of CSF Biomarkers

CSF was collected in AD and MCI patients by lumbar puncture between intervertebral spaces L3/L4 or L4/L5. After the centrifuge at 2000× *g* for 10 min, CSF was stored at −80 °C in polypropylene tubes. AD biomarkers were determined by enzyme-linked immunosorbent assays (ELISA) using the following assays: Aβ_1–42_ (Innotest β-amyloid1–42, Fujirebio, Tokyo, Japan), VILIP-1 (VILIP-1 Human ELISA, BioVendor, Brno, Czech Republic), p-tau_181_ (Innotest Phospho-Tau [181P], Fujirebio, Tokyo, Japan), p-tau_231_ (Tau [pT231] Phospho-ELISA Kit, Human, Thermo Fisher Scientific, Waltham, MA, USA), p-tau_199_ (TAU [pS199] Phospho-ELISA Kit, Human, Thermo Fisher Scientific), and t-tau (Innotest hTau AG, Fujirebio, Tokyo, Japan) (Table 1).

### 2.4. Determination of Polymorphisms

The salting-out method was used for the isolation of DNA from the peripheral blood [38]. In the 168 patients recruited at the University Hospital Center Zagreb, single nucleotide polymorphisms (SNPs) were determined by ABI Prism 7300 Real-Time PCR System apparatus (Applied Biosystems, Foster City, CA, USA), using the following TaqMan SNP Genotyping Assays (Applied Biosystems): *5HTR1B* rs13212041, *5HTR2A* rs6313 (T102C), *5HTR2C* rs3813929 (−759C/T), *5HTR6* rs1805054 (C267T), *APOE* rs7412, and rs429358. *APOE* SNPs were measured to determine *APOE* haplotypes (*APOE* ε2, ε3, and ε4) (rs429358 C allele and rs7412 C allele for ε4 variant, rs429358 T allele and rs7412 C allele for ε3 variant, and rs429358 T allele and rs7412 T allele for ε2 variant). SNPs were determined using Illumina genotyping platforms (CNV370v1, CNV370-Quadv3, and OmniExpressExome-8v1-2_A, Illumina, San Diego, CA, USA) in 2701 participants recruited from the “10,001 Dalmatians project”.

### 2.5. Statistical Analysis

Statistical analysis was performed with SPSS 19.0.1 (SPSS, Chicago, IL, USA). The level of statistical significance was set at α = 0.05. Levels of CSF biomarkers and scores on neuropsychological tests were compared between groups using the non-parametric Kruskal–Wallis test, while pairwise comparisons were conducted using a *post-hoc* non-parametric test (that corrects *p* values for multiple comparisons). The frequencies of different diagnoses and *APOE* genotypes among subjects with different *5HTR1B* rs13212041, *5HTR2A* rs6313 (T102C), *5HTR2C* rs3813929 (−759C/T), and *5HTR6* rs1805054 (C267T) genotypes and alleles were analyzed using a χ^2^-test, with applied correction for pairwise comparisons. When analyzing frequencies of different diagnoses among subjects with different *5HTR* genotypes, we included only HC of 70 years old and older (*n* = 461).

## 3. Results

### 3.1. Polymorphisms in 5HT Receptor Genes and CSF Biomarkers

**Figure 1:**
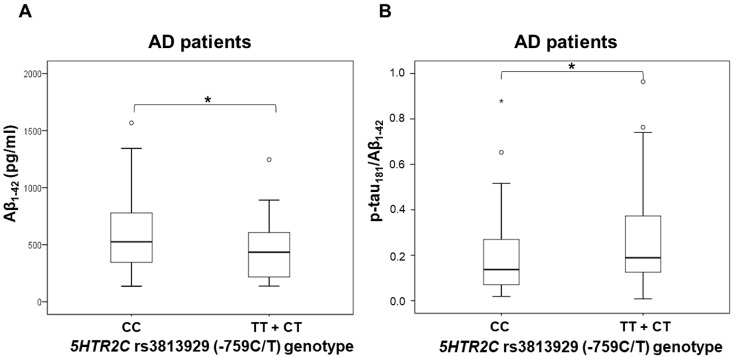
Levels of (**A**) Aβ_1–42_ and (**B**) p-tau_181_/Aβ_1–42_ ratio in AD patients with different *5HTR2C* rs3813929 (−759C/T) genotypes. * *p* < 0.05.

The CSF levels of Aβ_1–42_ were significantly decreased in AD patients with TT and CT genotypes compared to those with the CC *5HTR2C* rs3813929 (-759C/T) genotype (U = 1080, Z = −2.063, *p* = 0.039) (Figure 1). P-tau_181_/Aβ_1–42_ ratio was significantly increased in AD patients with TT and CT genotypes compared to those with the CC *5HTR2C* rs3813929 (-759C/T) genotype (U = 1056, Z = −2.121, *p* = 0.034) (Figure 1). There was no significant difference in the levels of CSF biomarkers (Aβ_1–42_, t-tau, p-tau_181_, p-tau_199_, p-tau_231_, VILIP-1, and p-tau_181_/Aβ_1–42_ ratio) between subjects with different *5HTR2A* rs6313 (T102C), *5HTR1B* rs13212041, and *5HTR6* rs1805054 (C267T) genotypes. No significant difference in t-tau, p-tau_181_, p-tau_199_, p-tau_231_, and VILIP-1 levels was observed between subjects with different *5HTR2C* rs3813929 (-759C/T) genotypes.

### 3.2. Polymorphisms in 5HT Receptor Genes, APOE Genotype, and AD Diagnosis

**Figure 2:**
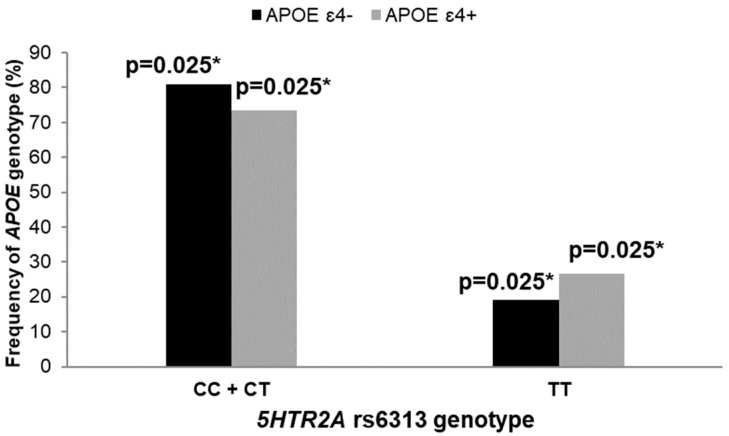
Frequency of *APOE* genotype in females younger than 65 years of age with different *5HTR2A* rs6313 genotypes. * *p* < 0.05.

We observed a significantly higher number of *APOE* ε4 allele carriers among female patients with the TT genotype compared to carriers of the CC and CT genotypes within the *5HTR2A* T102C polymorphism (χ^2^ = 7.453, df = 1; *p* = 0.006; Figure 2). This was also confirmed with logistic regression (β = 1.364, SE = 0.151, *p* = 0.040).

**Figure 3:**
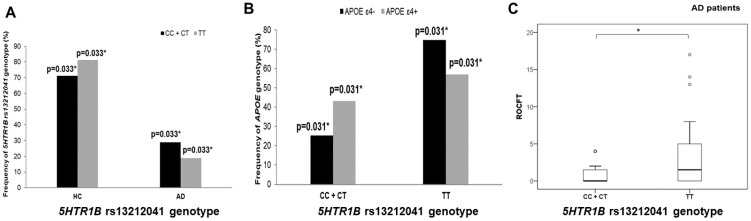
Participants carrying the C allele within *5HTR1B* rs13212041 polymorphism are (**A**) more represented among AD patients, (**B**) have higher frequency of *APOE* ε4 carriers (in males older than 65 years of age), and (**C**) show poorer performances on ROCFT test. * *p* < 0.05.

A significantly higher number of *APOE* ε4 allele carriers was also observed among male patients carrying the CC and CT genotypes compared to carriers of the TT genotype within the *5HTR1B* rs13212041 polymorphism (χ^2^ = 7.064, df = 1; *p* = 0.008; Figure 3). Additionally, a significantly higher number of individuals carrying the C allele within the *5HTR1B* rs13212041 polymorphism was observed among AD patients (χ^2^ = 6.973, df = 1; *p* = 0.008; Figure 3).

**Figure 4:**
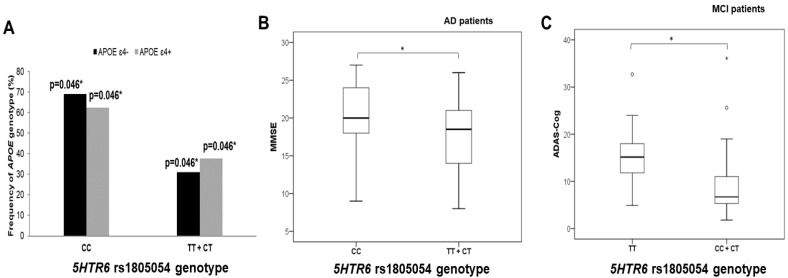
Participants carrying the T allele within *5HTR6* rs1805054 (C267T) polymorphism (**A**) have higher frequency of *APOE* ε4 carriers (in individuals younger than 65 years of age), (**B**) have poorer performances on MMSE (shown in AD patients), and (**C**) have poorer performances on ADAS–Cog (shown in MCI patients). * *p* < 0.05.

A significantly higher number of *APOE* ε4 allele carriers was also observed among individuals carrying the T allele within the *5HTR6* rs1805054 (C267T) polymorphism (χ^2^ = 6.425, df = 1; *p* = 0.011; Figure 4).

### 3.3. Polymorphisms in 5HT Receptors, Genes, and Neuropsychological Tests

AD patients carrying the C allele within the *5HTR1B* rs13212041 polymorphism had poorer performances on the ROCFT test (U = 216.5, Z = −2.106, *p* = 0.035; Figure 3).

Carriers of the T allele within the *5HTR6* rs1805054 had poorer performances on the ADAS–Cog (in MCI patients; U = 80.5, Z = −1.985, *p* = 0.046; Figure 4) and MMSE (in AD patients; t = −2.015, df = 108, *p* = 0.046; Figure 4). In contrast, AD patients carrying the CC genotype within the *5HTR6* rs1805054 had poorer performances on the VAT test compared to TT and CT genotype carriers (U = 223, Z = −2.224, *p* = 0.026).

## 4. Discussion

The serotonergic system is highly affected in AD [1,2,3,4]. The main serotonergic nucleus that projects throughout the cortex, the dorsal raphe nucleus (DRN, B7-B9), is affected early by AD pathological changes, with neurofibrillary pathology in all of Braak stage I and more than 20% of Braak stage 0 cases [16]. In addition, altered activity of DRN neurons due to the accumulation of AD pathological changes is thought to cause BPSD in early AD [39,40,41], which is compatible with a reported decrease in the serotonergic innervation of the hippocampus and neocortex [2,11,12,13].

Changes in serotonergic receptors are also detected in AD. Loss of 5HT_1B/1D_ and 5HT_6_ receptors was observed in the frontal and temporal cortex of AD patients [17]. Reduction in 5HT_1A_ receptor binding [18] and loss of 5HT_2A_ receptors [19] was observed in the AD brain using positron emission tomography (PET) imaging. Additionally, reduced binding to the 5HT_1A_ receptor in the hippocampus and temporal neocortex, respectively, correlates with cognitive decline [42], and aggressive behavior [43]. Activation of 5HT_4_, 5HT_6_, and 5HT_7_ receptors in experimental models of AD resulted in a decrease in Aβ content [44,45,46,47], while injections of Aβ in the hippocampi of mouse models of AD [48,49] leads to a reduction in 5HT_2A_ receptor expression. Interestingly, serotonergic receptors are potential targets for AD therapeutics [4] as their activation affects signaling pathways involved in the production of Aβ and hyperphosphorylated tau protein [3]. Activation of 5HTR_4_, 5HTR_6_, and 5HTR_7_ results in reduced production of Aβ (for details see [45]). Additionally, the activation of various 5HT receptors can modify tau phosphorylation. For example, the activation of 5HTR_1A_ activates the phosphoinositide 3-kinase (PI3K), phosphoinositide-dependent kinase (PDK), and protein kinase B (AKT) cascade. AKT phosphorylates and consequently inactivates glycogen synthase kinase-3 (GSK3) that phosphorylates tau protein. 5HTR_2_ could modulate GSK3 phosphorylation through protein kinase C (PKC) [50] and β-arrestin-mediated signaling [51], while 5HTR_4_, 5HTR_6_, and 5HTR_7_ could modulate GSK3 phosphorylation through protein kinase A (PKA) [50]. Several studies also observed an association between APOE and 5HT receptors. Shinohara et al. showed that a 5HTR_3_ antagonist (ondansetron) increases apoE secretion through the liver X receptor (LXR) and ATB-binding cassette protein A1 (ABCA1) pathway [52]. Additionally, Chhibber and Zhao observed a significant difference in 5HT receptor expression levels in mice carrying different *ApoE* genotypes [53]. Specifically, 5HTR2A protein expression levels were higher in the cortexes of mice with human *APOE4* gene-targeted replacement than in mice with *ApoE2* and *ApoE3* genotypes. However, 5HTR_1A_ protein levels did not differ among mice with different *ApoE* genotypes [53].

In this study, we assessed whether the levels of CSF AD biomarkers, scores on neuropsychological tests, and genetic biomarkers of AD (*APOE* haplotype) differed between patients with various *5HTR1B* rs13212041, *5HTR2A* rs6313 (T102C), *5HTR2C* rs3813929 (−759C/T), and *5HTR6* rs1805054 (C267T) polymorphisms. We observed a significantly higher number of *APOE* ε4 allele carriers among individuals carrying the TT genotype within the *5HTR2A* T102C polymorphism, the C allele within the *5HTR1B* rs13212041 polymorphism, and the T allele within the *5HTR6* rs1805054 (C267T) polymorphism. Additionally, individuals carrying the C allele within the *5HTR1B* rs13212041 polymorphism were significantly more represented among AD patients and had poorer performances on the ROCFT test. Carriers of a T allele within the *5HTR6* rs1805054 had poorer performances on the MMSE and ADAS–Cog, while a significant decrease in the levels of CSF Aβ_1–42_ and an increase in the p-tau_181_/Aβ_1–42_ ratio was observed in carriers of a T allele in the *5HTR2C* rs3813929 (−759C/T) polymorphism.

Our study shows that AD patients carrying a T allele in the *5HTR2C* rs3813929 (−759C/T) polymorphism have pathological CSF Aβ_1–42_ levels. The *5HTR2C* -759C/T polymorphism did not affect the expression levels of the 5HT_2C_ receptor [54], and the effect of the *5HTR2C* −759C/T polymorphism on 5HT_2C_ receptor expression in different tissues is also not documented in the Genotype-Tissue Expression (GTEx) project database [55]. However, Buckland et al. observed that the C allele within the *5HTR2C* −759C/T polymorphism shows less transcriptional activity compared to the T allele [56]. The association of the *5HTR2C* -759C/T polymorphism with AD was not previously reported. However, in vitro [57] and in vivo [58] experiments showed that 5HT_2C_ receptor activation stimulates the release of soluble amyloid precursor protein (sAPP). Our study reveals that carriers of the T allele in the *5HTR2C* rs3813929 (-759C/T) polymorphism have pathological CSF Aβ_1–42_ levels, and Buckland et al.’s study showed that the T allele within the *5HTR2C* -759C/T polymorphism increases transcriptional activity [56]. Thus, it is possible that this polymorphism indirectly affects the release of sAPP and the amount of produced Aβ_1–42_.

Additionally, this study shows that carriers of the T allele within the *5HTR6* rs1805054 (C267T) polymorphism have poorer performances on the MMSE and ADAS–Cog tests and that a higher number of *APOE* ε4 allele carriers is observed among these individuals. The *5HTR6* C267T polymorphism does not involve an amino acid change, but this silent mutation could affect the splicing process [59]. According to the GTEx portal [55], this SNP significantly affects the expression levels of the 5HT_6_ receptor, with carriers of the T allele within the *5HTR6* rs1805054 (C267T) polymorphism having a lower expression of 5HT_6_ receptor mRNA in whole blood. The *5HTR6* C267T polymorphism was previously associated with AD, albeit with conflicting results. Tsai et al. observed a higher frequency of the CC *5HTR6* C267T genotype in AD patients compared to controls [60], while Kan et al. observed an increased number of CT *5HTR6* C267T heterozygotes among AD patients [61]. Moreover, other authors did not find an association between *5HTR6* C267T polymorphism and AD [59,62,63]. Our study did not observe a difference in the distribution of *5HTR6* C267T genotypes between AD patients and controls, but this SNP elucidated an association between neuropsychological and genetic biomarkers of AD. The association between the *5HTR6* C267T polymorphism and cognitive decline in AD observed in this study is not surprising given that several studies elucidated an association between this receptor and AD (reviewed in [64]). In fact, the potential of 5HT_6_ receptor antagonists as therapeutics for AD has been tested in a number of studies [65].

Our study also revealedan association of the C allele within the *5HTR1B* rs13212041 polymorphism with genetic and neuropsychological biomarkers of AD and AD diagnosis that has not been previously associated with AD. The effect of the *5HTR1B* rs13212041 polymorphism on 5HT_1B_ receptor expression in different tissues is also not documented in the GTEx portal [55], although Jensen et al. showed that carriers of the T allele within the *5HTR1B* rs13212041 polymorphism show reduced 5HTR_1B_ expression compared to carriers of the C allele [66].

Finally, we observed a significantly higher number of *APOE* ε4 allele carriers among individuals carrying the TT genotype within the *5HTR2A* T102C polymorphism. According to the GTEx portal [55], this SNP does not affect the levels of 5HTR2A in the brain, although it significantly affected 5HTR2A expression in testes, muscles, and aortae. This polymorphism is located within the first exon of the *5HTR2A* gene and, being near the promoter region, could be involved in gene regulation [67]. Li et al. recently showed that the *5HTR2A* T102C polymorphism increases the risk of AD [68]. Interestingly, the *5HTR2A* T102C polymorphism also showed an association with BPSD in AD [21,22,23,24,25,26], although inconsistently among studies [69,70,71,72].

## 5. Conclusions

In this study, we observed differences in the distribution of 5HT receptor gene genotypes and *APOE* genotypes between male and female participants. Gender difference in the distribution of both *APOE* genotypes and 5HT receptor gene genotypes was previously reported [73,74]. Namely, it was shown that elderly female *APOE* ε4 carriers are at higher risk of developing AD [75], show stronger cognitive decline [76], weaker brain connectivity (detected using functional magnetic resonance imaging (fMRI) in the precuneus and posterior cingulate cortex) [73], and lower brain metabolism [77] than males. In contrast, Cacciottolo et al. showed that elderly males diagnosed with AD or MCI carrying the *APOE* ε4 allele had a higher risk of brain microbleeds compared to females with the same genotype and condition [78]. Interestingly, a similar sex-dependent relationship between *HTR2C* gene variants and suicidal behavior [79] and *HTR1B* polymorphisms and schizophrenia [80] has been reported.

Our data reveal that all four analyzed polymorphisms of 5HT receptor genes had an association with either genetic, CSF, or neuropsychological biomarkers of AD. As such, considering the early involvement of the serotonergic systems in the progression of AD, these polymorphisms represent interesting diagnostic and therapeutic targets and deserve further investigation as potential early genetic biomarkers of AD.

## Figures and Tables

**Table 1:** Demographic data and biomarkers in different cohorts.

		AD	MCI	HC
Measured biomarkers	CSF	+	+	-
	Genetic	+	+	+
	Neuropsychological	+	+	−
*n*		115	53	2701
Age	Median	73	70	55
	(25–75th percentile)	(67–77)	(60–75)	(43–66)
Sex	F/M	62/53	27/26	1714/987
MMSE	Mean ± SD	19.6 ± 5.2	25.1 ± 3	−
Aβ_1–42_ (pg/mL)	Mean ± SD	536.9 ± 296.9	723.4 ± 371.9	−
T-tau (pg/mL)		520.0 ± 394.4	246.4 ± 158.0	−
p-tau_181_ (pg/mL)		80.0 ± 47.8	57.6 ± 30.9	−
p-tau_199_ (pg/mL)		4.4 ± 3.5	3.4 ± 2.4	−
p-tau_231_ (U/mL)		3.9 ± 5.5	1.8 ± 3.2	−
VILIP-1 (pg/mL)		138.3 ± 88.5	94.9 ± 78.1	−

Aβ_1–42_, amyloid β_1–42_; AD, Alzheimer’s disease; CSF, cerebrospinal fluid; F, female; HC, healthy controls; M, male; MCI, mild cognitive impairment; MMSE, Mini-Mental State Examination; p-tau_181_, tau phosphorylated at Thr 181; p-tau_199_, tau phosphorylated at Ser 199; p-tau_231_, tau phosphorylated at Thr 231; t-tau, total tau; VILIP-1, visinin-like protein 1.

**Table 2:** Number of *APOE* and *5HTR* genotypes in different cohorts.

		AD	MCI	HC
*APOE*	ε2ε2			10
	ε3ε2	9	1	252
	ε3ε3	58	36	1966
	ε4ε3	36	14	421
	ε4ε4	7	2	28
	ε4ε2	5		24
*5HTR2C* rs3813929 (−759C/T)	CC	79	37	
	CT	24	12	−
	TT	12	4	
*5HTR2A* rs6313	CC	40	18	911
	CT	56	27	1267
	TT	19	8	523
*5HTR1B* rs13212041	CC	6	1	87
	CT	38	16	648
	TT	71	36	1966
*5HTR6* rs1805054 (C267T)	CC	59	28	1834
	CT	33	18	768
	TT	2	1	99

5HTR2A, 5-hydroxytryptamine receptor 2A; 5HTR1B, 5-hydroxytryptamine receptor 1B; 5HTR2C, 5-hydroxytryptamine receptor 2C; 5HTR6, 5-hydroxytryptamine receptor 6; AD, Alzheimer’s disease; APOE, apolipoprotein E; HC, healthy controls; MCI, mild cognitive impairment.

## Data Availability

All the data reported are available on request from the corresponding author.

## Abbreviations

5HT: Serotonin; *5HTR*, gene for 5HT receptor; Aβ, amyloid β; ABCA1, ATB-binding cassette protein A1; AD, Alzheimer’s disease; ADAS–Cog, Alzheimer’s Disease Assessment Scale–cognitive subscale; AKT, protein kinase B; APOE, apolipoprotein E; BPSD, behavioral and psychological symptoms of dementia; CDT, Clock Drawing Test; CSF, cerebrospinal fluid; DRN, dorsal raphe nucleus; ELISA, enzyme-linked immunosorbent assays; fMRI, functional magnetic resonance imaging; GSK3, glycogen synthase kinase-3; LP, lumbar puncture; LXR, liver X receptor; MCI, mild cognitive impairment; MMSE, Mini-Mental State Examination; NIA–AA, National Institutes on Aging–Alzheimer’s Association; PDK, phosphoinositide-dependent kinase; PET, positron emission tomography; PI3K, phosphoinositide 3-kinase; PKA, protein kinase A; PKC, protein kinase C; p-tau_181_, tau phosphorylated at Thr 181; p-tau_199_, tau phosphorylated at Ser 199; p-tau_231_, tau phosphorylated at Thr 231; ROCFT, Rey–Osterrieth complex figure test; sAPP, soluble amyloid precursor protein; SNP, single nucleotide polymorphisms; t-tau, total tau; VAT, Visual Association Test; VILIP-1, visinin-like protein 1.
